# Data on step-by-step atomic force microscopy monitoring of changes occurring in single melanoma cells undergoing ToF SIMS specialized sample preparation protocol

**DOI:** 10.1016/j.dib.2016.07.052

**Published:** 2016-08-03

**Authors:** J. Bobrowska, J. Pabijan, J. Wiltowska-Zuber, B.R. Jany, F. Krok, K. Awsiuk, J. Rysz, A. Budkowski, M. Lekka

**Affiliations:** a The Institute of Nuclear Physics, Polish Academy of Sciences, Radzikowskiego 152, 31-342 Cracow, Poland; b Marian Smoluchowski Institute of Physics, Jagiellonian University, Lojasiewicza 11, 30-348 Cracow, Poland

**Keywords:** ToF SIMS, Single cells preparation, AFM imaging

## Abstract

Data included in this article are associated with the research article entitled ‘Protocol of single cells preparation for time-of-flight secondary ion mass spectrometry’ (Bobrowska et al., 2016 in press) [1]. This data file contains topography images of single melanoma cells recorded using atomic force microscopy (AFM). Single cells cultured on glass surface were subjected to the proposed sample preparation protocol applied to prepare biological samples for time-of-flight secondary ion mass spectrometry (ToF SIMS) measurements. AFM images were collected step-by-step for the single cell, after each step of the proposed preparation protocol. It consists of four main parts: (i) paraformaldehyde fixation, (ii) salt removal, (iii) dehydrating, and (iv) sample drying. In total 13 steps are required, starting from imaging of a living cell in a culture medium and ending up at images of a dried cell in the air. The protocol was applied to melanoma cells from two cell lines, namely, WM115 melanoma cells originated from primary melanoma site and WM266-4 ones being the metastasis of WM115 cells to skin.

**Specifications Table**

**Table:** 

Subject area	*Physics, Chemistry, Biology*
More specific subject area	*Biophysics, Biochemistry*
Type of data	*Figures*
How data was acquired	*Atomic force microscope (AFM): XE120 Park Systems. Surface images (topography and deflection modes and 3D representation of topography) recorded by Xe120 system (saved originally in tiff format)*
Data format	*Analyzed*
Experimental factors	*Living melanoma cells were cultured on the glass coverslips, placed in a Petri dish, and immersed in a culture medium. Such samples were further used for the AFM measurements.*
Experimental features	*AFM images of surface topography of single melanoma cells were measured after each step of the applied preparation protocol. The AFM topography was acquired for the same cell.*
Data source location	*Poland, Cracow, Latitude:50° 3.879006׳ Longitude:19° 56.698794׳*
Data accessibility	*Data are provided with this article*

**Value of the data**

• The data presented here essentially show changes on a cell surface upon application of the ToF SIMS specific sample preparation protocol.
• Recorded images enable to estimate the effect of fixation, salt removal, dehydration, and air-drying on surface topography and morphology of single cells.
• The proposed protocol for sample preparation can be applied to prepare single cells cultured on bare silicon surface for ToF SIMS measurements delivering mass spectra of single cells.

## 1 Data

**Fig. 1.1:**
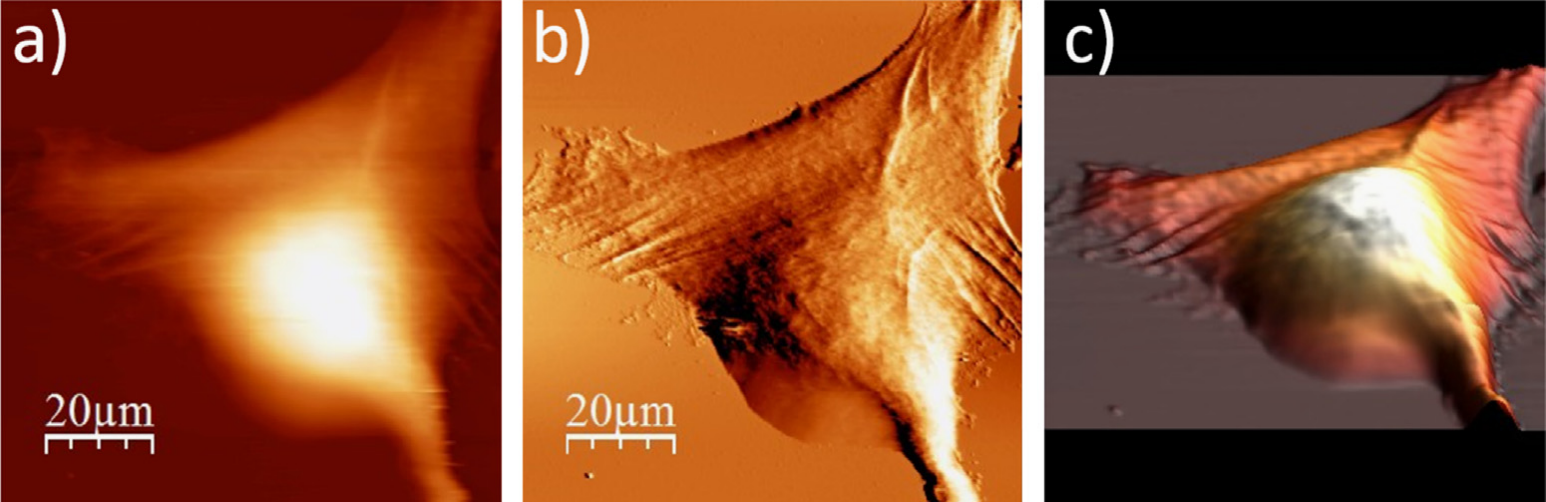
Cell surface of a single alive WM115 cell measured in the culture medium: (a) AFM topography, (b) deflection (“error”) images, and (c) 3D representation of a single alive melanoma WM115 cell measured in the culture medium.

**Fig. 1.2:**
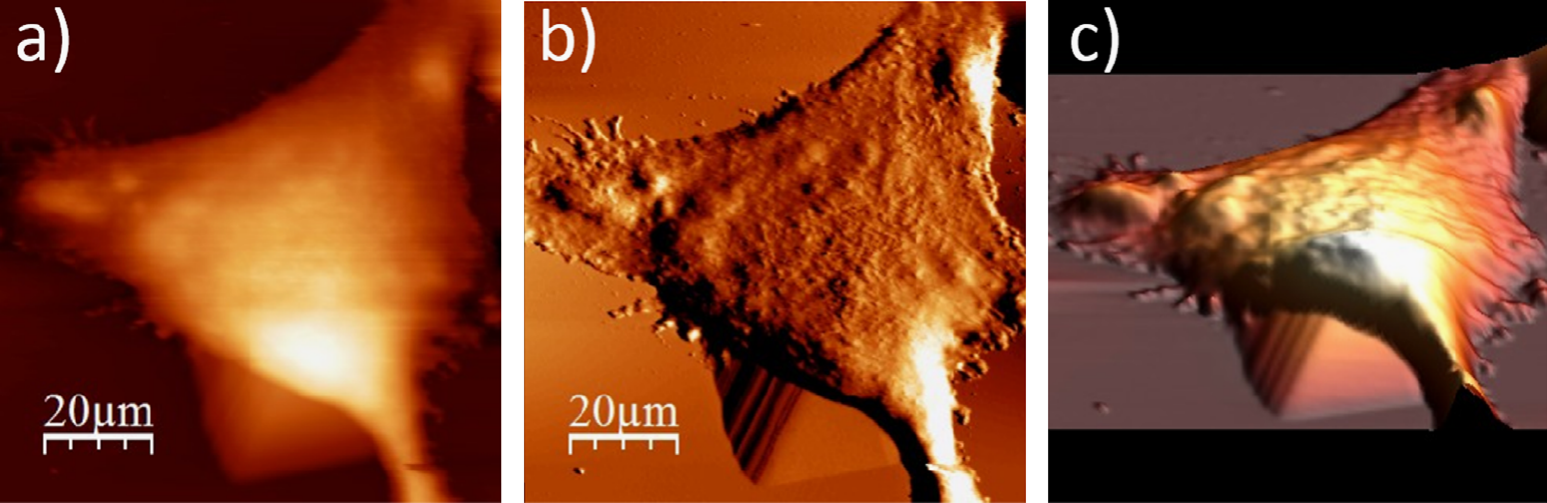
WM115 cell surface changes after fixation with paraformaldehyde: (a) AFM topography, (b) deflection (“error”) images, and (c) 3D representation of WM115 cell fixed with 3.7% of paraformaldehyde dissolved in the phosphate buffered saline (PBS) for 20 minutes, measured in the PBS buffer.

**Fig. 1.3:**
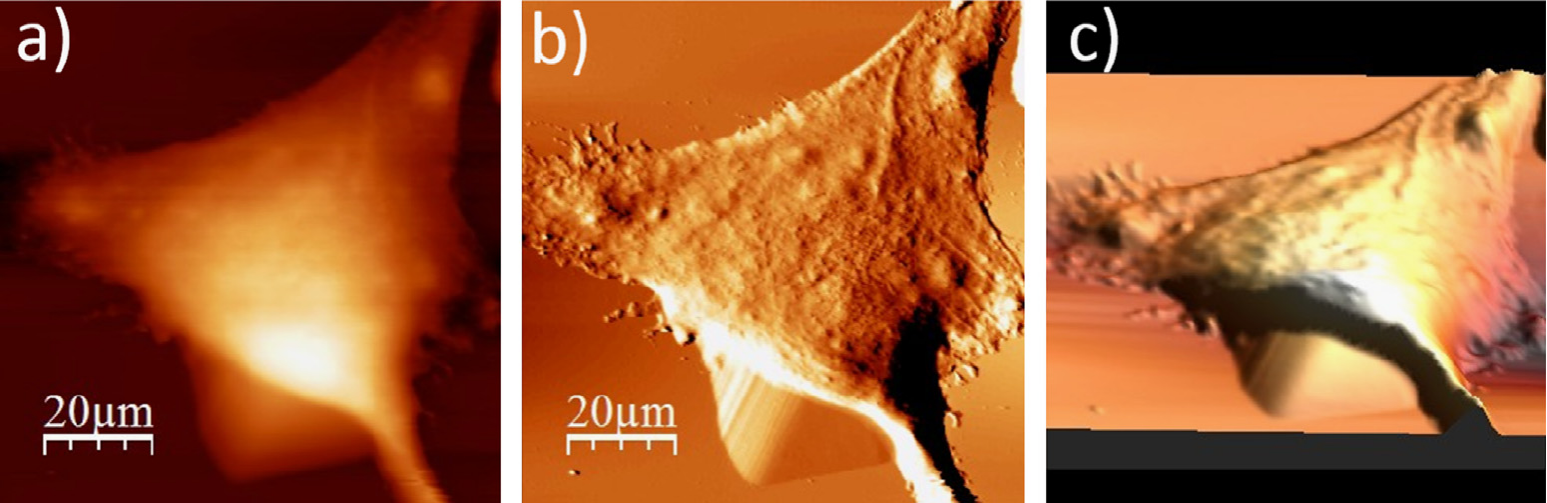
Surface of a single WM115 melanoma cell after rinsing in 50% PBS buffer: (a) AFM topography, (b) deflection (“error”) images, and (c) 3D representation of WM115 cell rinsed with 50% PBS buffer, measured in the 50% PBS buffer (1:1, PBS:water).

**Fig. 1.4:**
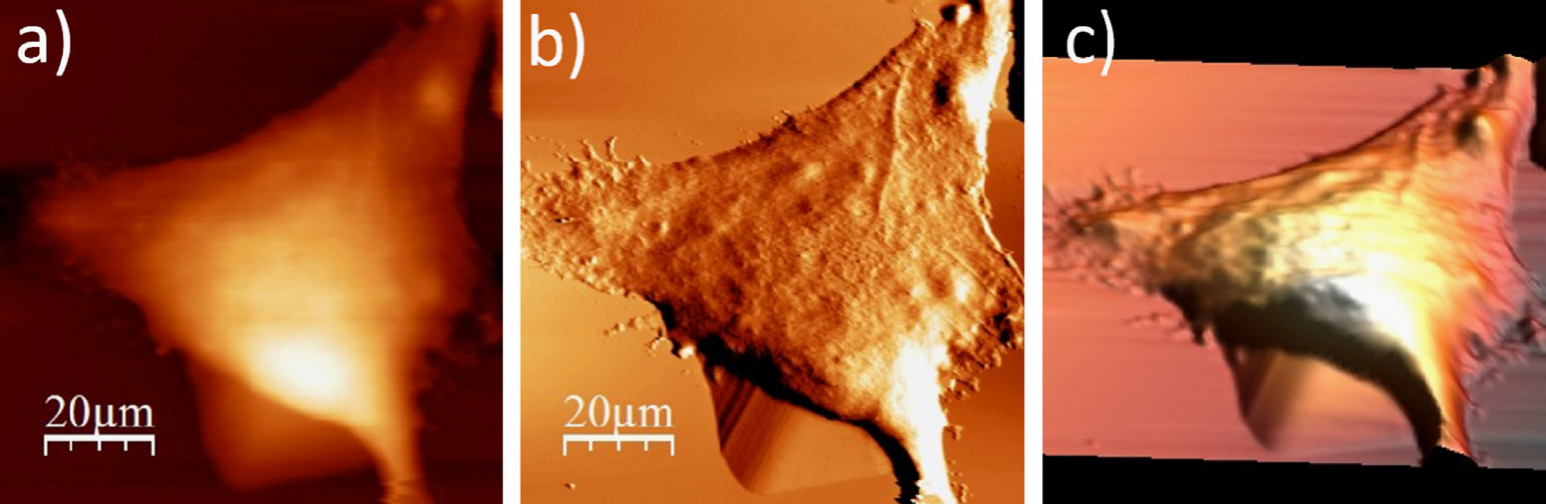
Surface of a single WM115 melanoma cell after rinsing in 25% PBS buffer: (a) AFM topography, (b) deflection (“error”) images, and (c) 3D representation of WM115 cell rinsed with 25% PBS buffer, measured in the 25% PBS buffer (1:4, PBS:water).

**Fig. 1.5:**
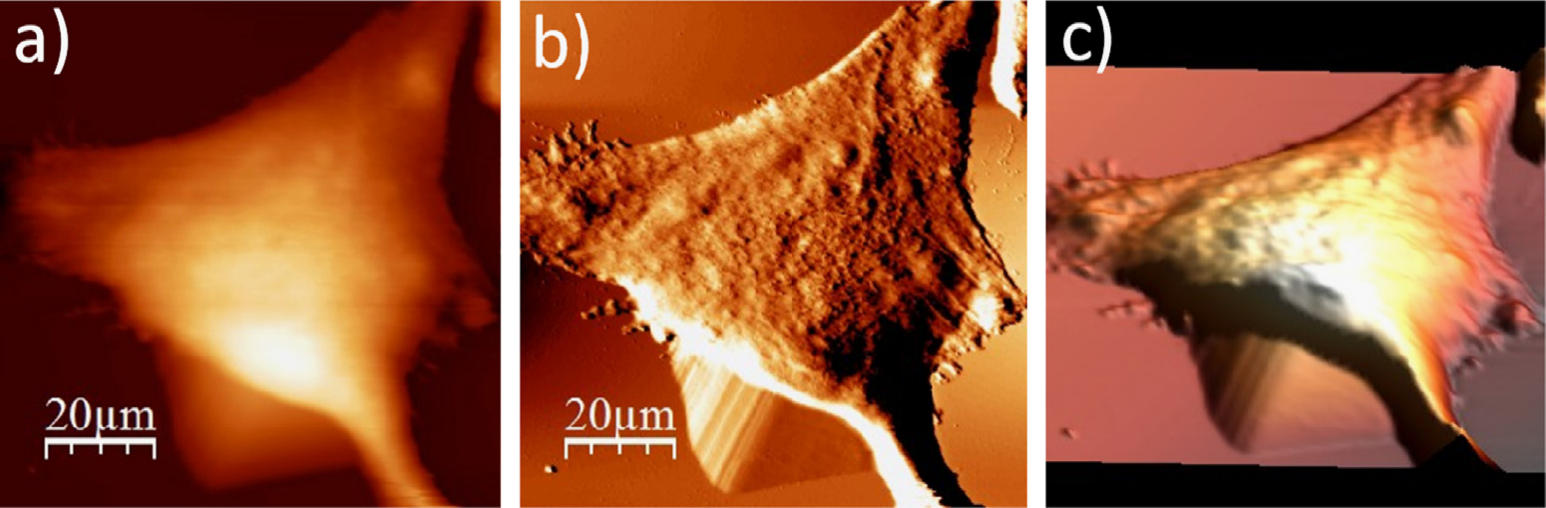
Surface of a single WM115 melanoma cell after rinsing with deionized water: (a) AFM topography, (b) deflection (“error”) images, and (c) 3D representation of WM115 cell rinsed with deionized water (Cobrabid purification system, 0.08 µS).

**Fig. 1.6:**
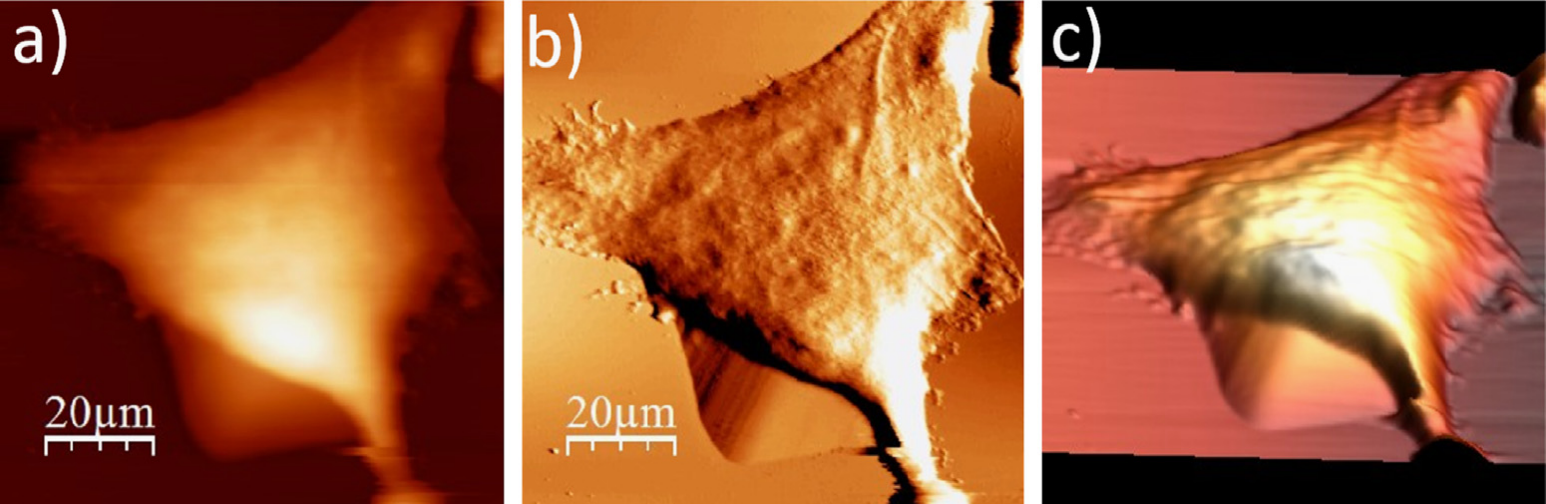
Surface of a single WM115 melanoma cell after rinsing with 40% alcohol: (a) AFM topography, (b) deflection (“error”) images, and (c) 3D representation of WM115 cell rinsed with 40% aqueous solution of ethyl alcohol.

**Fig. 1.7:**
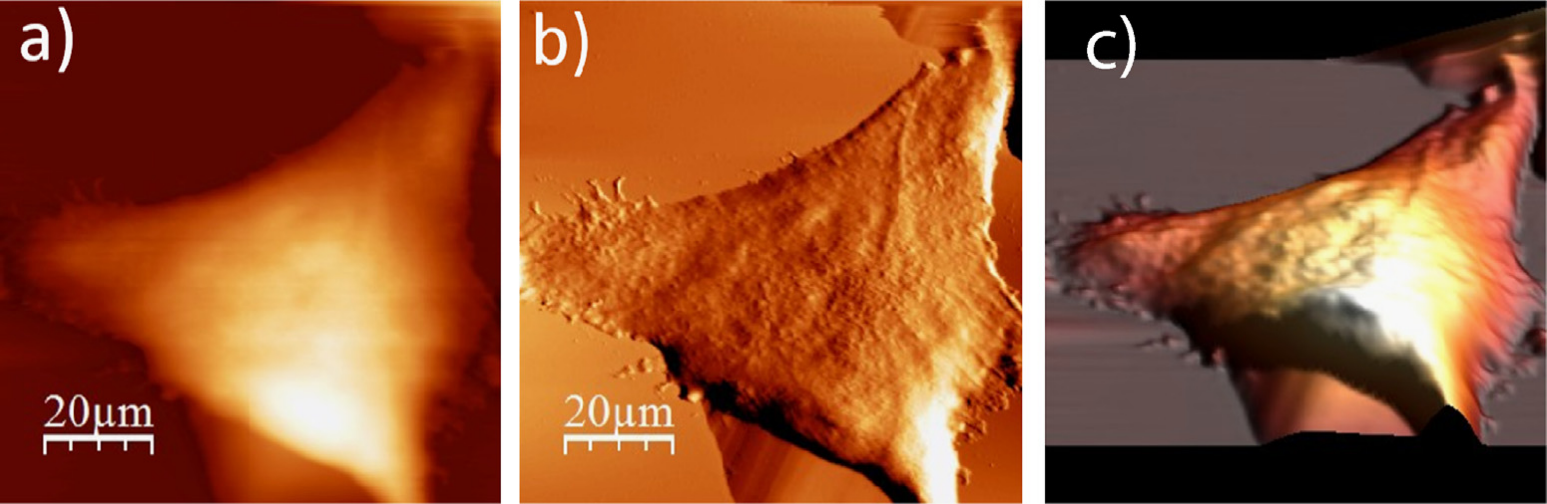
Surface of a single WM115 melanoma cell after rinsing with 50% alcohol: (a) AFM topography, (b) deflection (“error”) images, and (c) 3D representation of WM115 cell rinsed with 50% aqueous solution of ethyl alcohol.

**Fig. 1.8:**
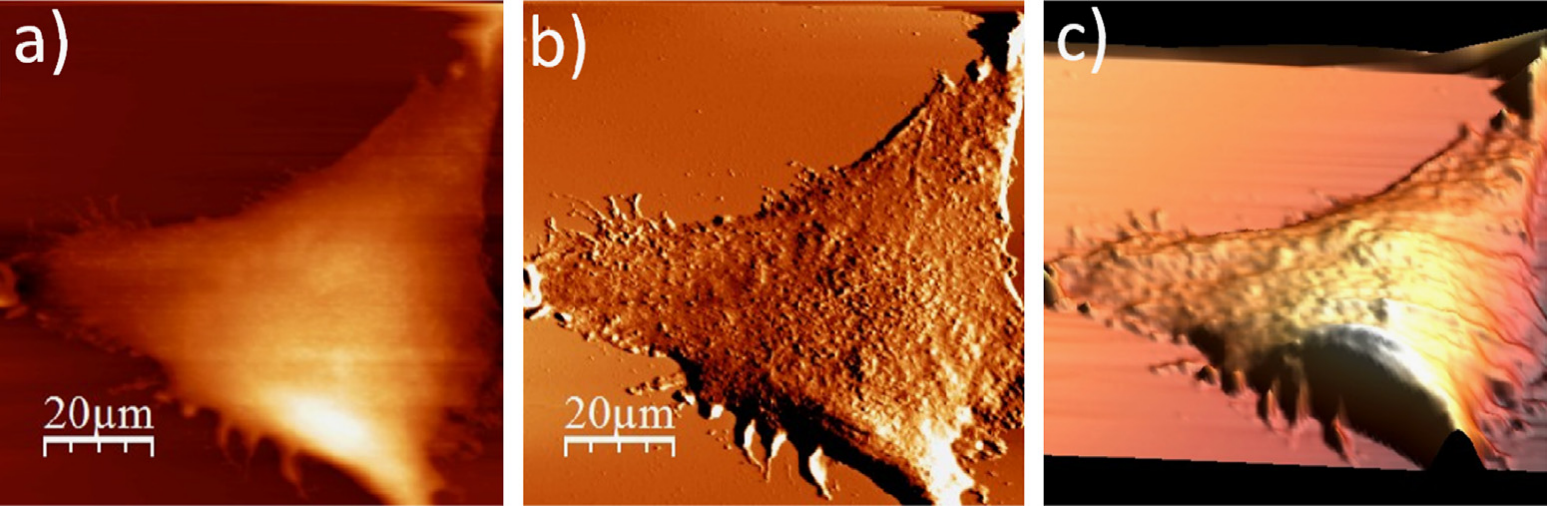
Surface of a single WM115 melanoma cell after rinsing with 60% alcohol: (a) AFM topography, (b) deflection (“error”) images, and (c) 3D representation of WM115 cell rinsed with 60% aqueous solution of ethyl alcohol.

**Fig. 1.9:**
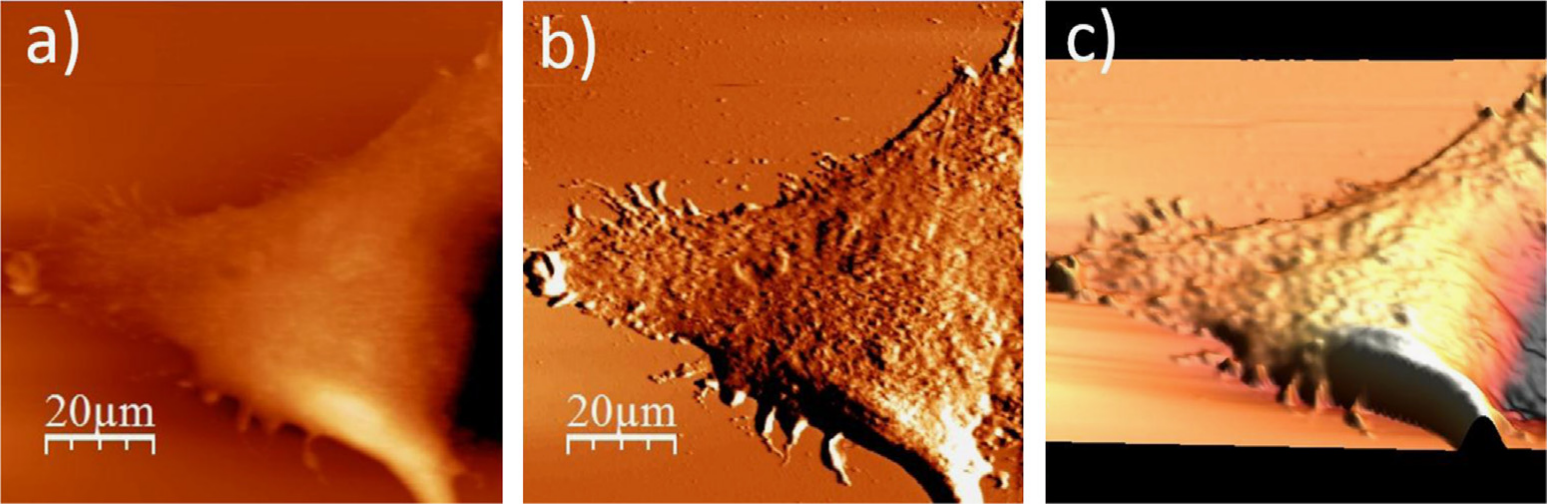
Surface of a single WM115 melanoma cell after rinsing with 70% alcohol: (a) AFM topography, (b) deflection (“error”) images, and (c) 3D representation of WM115 cell rinsed with 70% aqueous solution of ethyl alcohol.

**Fig. 1.10:**
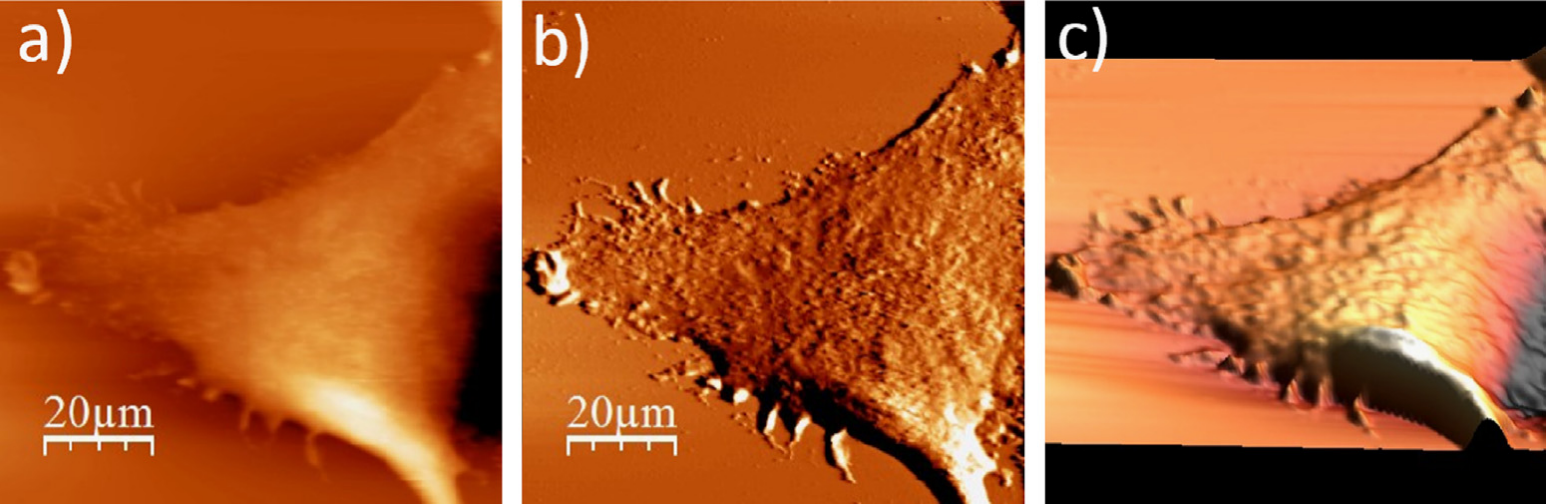
Surface of a single WM115 melanoma cell after rinsing with 80% alcohol: (a) AFM topography, (b) deflection (“error”) images, and (c) 3D representation of WM115 cell rinsed with 80% aqueous solution of ethyl alcohol.

**Fig. 1.11:**
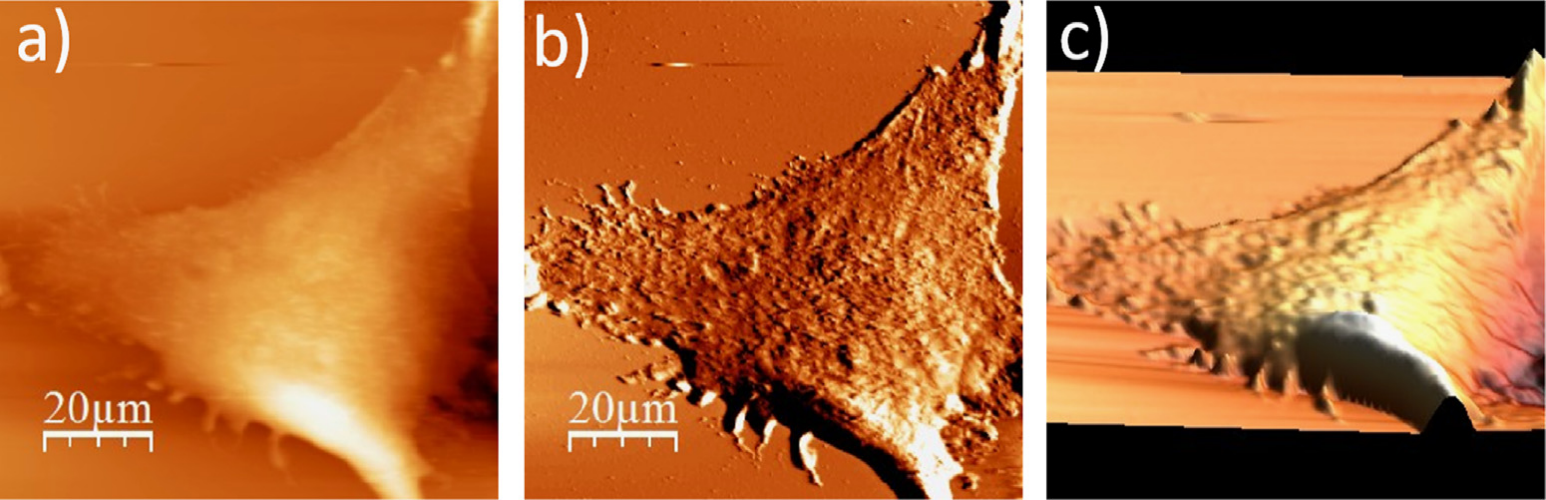
Surface of a single WM115 melanoma cell after rinsing with 90% alcohol: (a) AFM topography, (b) deflection (“error”) images, and (c) 3D representation of WM115 cell rinsed with 90% aqueous solution of ethyl alcohol.

**Fig. 1.12:**
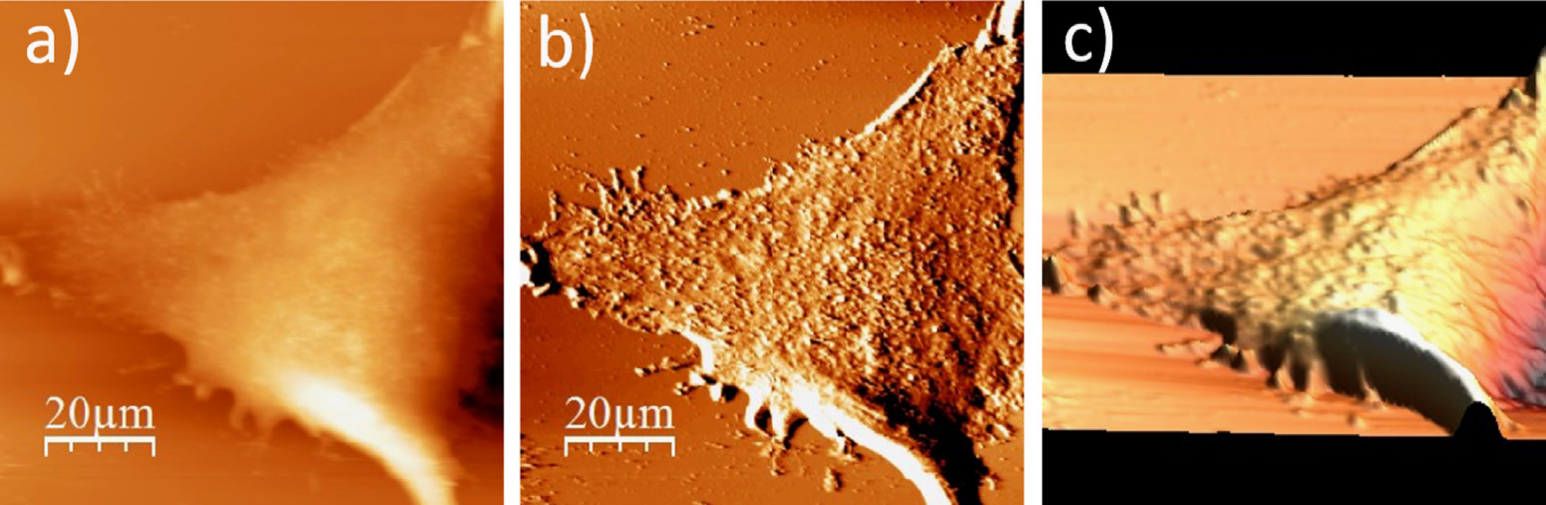
Surface of a single WM115 melanoma cell after rinsing with anhydrous alcohol: (a) AFM topography, (b) deflection (“error”) images, and (c) 3D representation of WM115 cell rinsed with anhydrous alcohol (99. 8%).

**Fig. 1.13:**
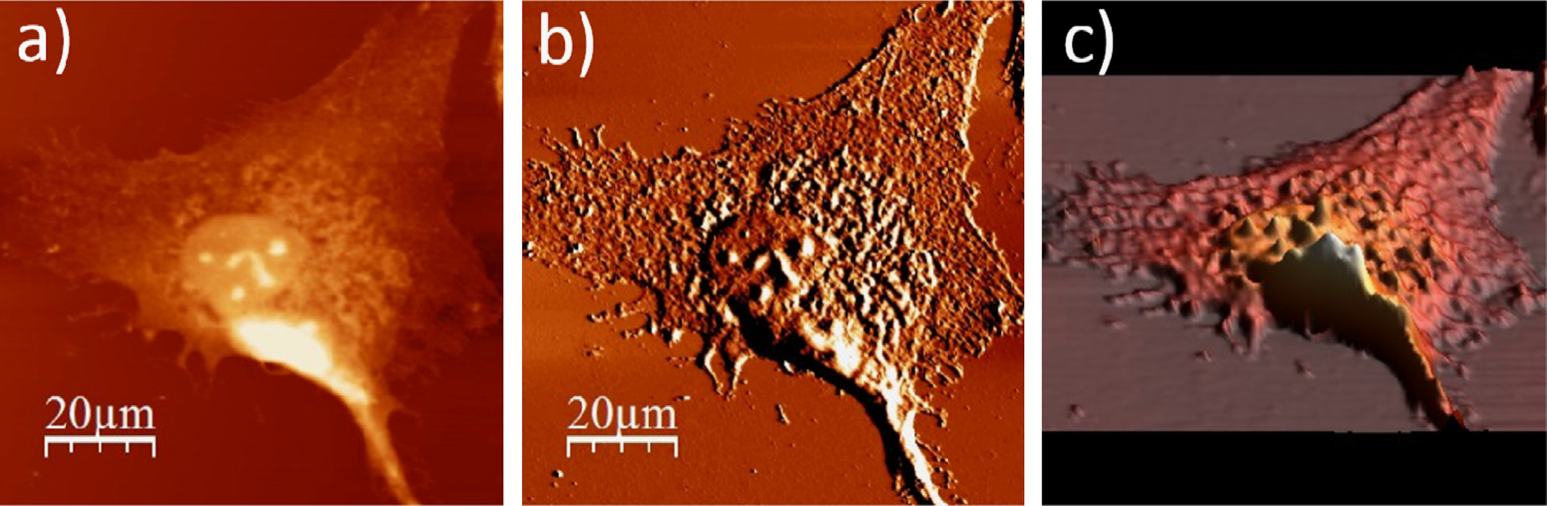
Surface of a dried single WM115 melanoma cell: (a) AFM topography, (b) deflection (“error”) images, and (c) 3D representation of a dried WM115 cell.

The data presented below, show an exemplary sequence of the surface topography and deflection (“error”) images (a & b) recorded using an atomic force microscope (model: Xe120, from Park Systems, Korea) for a single cell after each step of the applied ToF SIMS specific sample preparation protocol, accompanied by a 3D representation of a cell surface (c). The protocol involve four main stages, i.e. fixation, salt removal, dehydration and air-drying. The recorded data show changes on the surface of single WM115 cell. The use of the proposed sample preparation protocol enables to record mass spectra (ToF SIMS) on single cells [1], [2] (Fig. 1.1, Fig. 1.2, Fig. 1.3, Fig. 1.4, Fig. 1.5, Fig. 1.6, Fig. 1.7, Fig. 1.8, Fig. 1.9, Fig. 1.10, Fig. 1.11, Fig. 1.12, Fig. 1.13).

The AFM images presented below, show surface changes of single WM266-4 cells acquired after each step of the ToF SIMS specialized sample preparation protocol. The presented protocol enables to record mass spectra (ToF SIMS) on single cells [1], [2] (Fig. 2.1, Fig. 2.2, Fig. 2.3, Fig. 2.4, Fig. 2.5, Fig. 2.6, Fig. 2.7, Fig. 2.8, Fig. 2.9, Fig. 2.10, Fig. 2.11, Fig. 2.12, Fig. 2.13).

## 2 Experimental design, materials and methods

### 2.1 Cell culture

WM115 (skin primary tumor site) and WM266-4 (metastasis to skin) melanoma cells were cultured in the RPMI-1640 medium (Sigma-Aldrich) supplemented with 10% fetal bovine serum (FBS, Sigma-Aldrich). The cells were grown in 25 cm^2^ culture flasks (Sarstedt) in the incubator (NuAire) (37 °C and a 95% air/5% CO_2_ atmosphere). After several passages (6–8), cells were seeded on clean and sterile glass coverslips or silicon substrates, placed in the Petri dishes (Sarstedt), and further cultured for 48 h in the corresponding media and culture conditions. For the AFM topography measurements cells were cultured directly on a bare glass coverslips while for ToF SIMS experiments they were grown on bare silicon substrates.

### 2.2 ToF SIMS specific protocol

The protocol for single cell preparation for ToF SIMS experiments follows through the following steps (step number correlates with image number):

1. Living cell cultured on bare silicon surface (for AFM measurements – on glass surface).

2. Fixation with paraformaldehyde.

3. Rinsing with 50% phosphate buffered saline solution (PBS).

4. Rinsing with 25% PBS.

5. Rinsing with deionized water.

6. Washing with 40% ethyl alcohol aqueous solution.

7. Washing with 50% ethyl alcohol aqueous solution.

8. Washing with 60% ethyl alcohol aqueous solution.

9. Washing with 70% ethyl alcohol aqueous solution.

10. Washing with 80% ethyl alcohol aqueous solution.

11. Washing with 90% ethyl alcohol aqueous solution.

12. Washing with anhydrous ethyl alcohol.

13. Drying.

### 2.3 Atomic force microscopy

Topography of cells’ surface was recorded after each step of the sample preparation protocol using a commercial AFM set-up working in contact mode (model Xe120, Park Systems, Korea). Images were obtained using V-shaped silicon nitride cantilevers, characterized by nominal spring constant of 0.03 N/m (PNP-TR-customized, Nanoworld). The set point ranged from 0.2 nN to 0.8 nN (adjusted during the topography acquisition) while the scan rate range was set between 0.3 Hz and 1 Hz depending on the scan size. In the presented measurements, glass coverslips with living cells, placed in the Petri dish and immersed in a culture medium (RPMI-1640 culture media supplemented with 1% HEPES (pH 7.5)), were mounted on the top of a piezoelectric scanner. First, an image of a single alive cell was recorded in the culture medium. Then, images of the same cell were collected after each steps of the proposed protocol for single cells’ preparation for ToF SIMS spectroscopy. In total, a sequence of thirteen images was collected. Images were analyzed using XEI (dedicated AFM software provided by Park Systems) and WSxM 5.0 software [3], only the contrast and slope of the images were adjusted when necessary.

## Figures and Tables

**Fig. 2.1:**
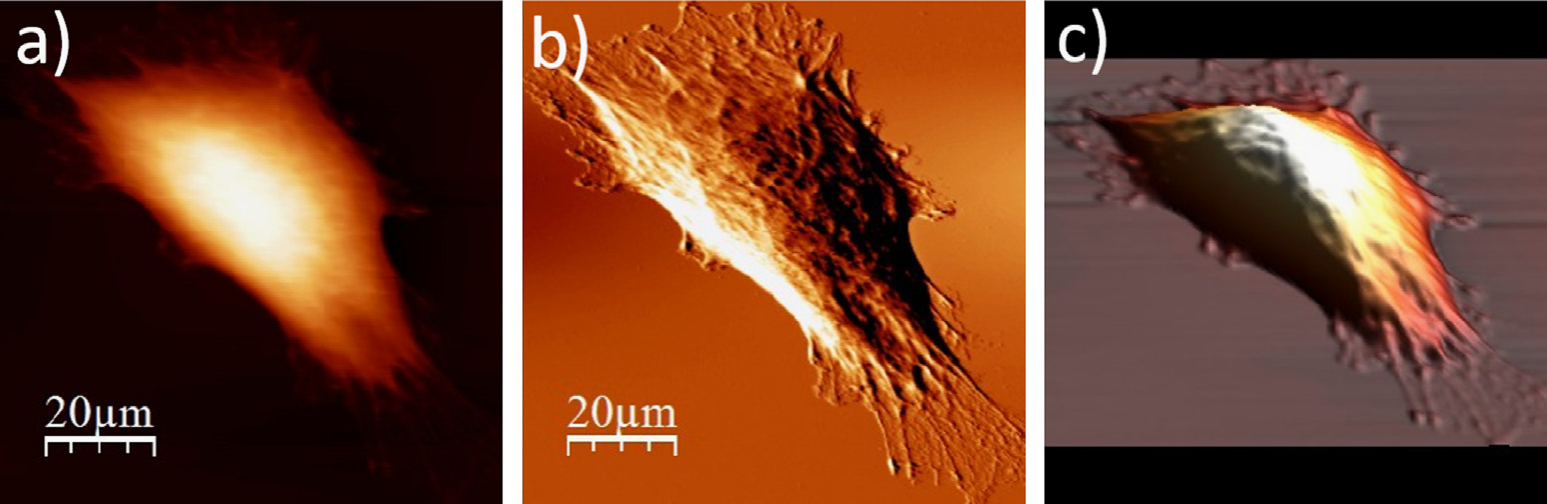
Cell surface of a single alive WM266-4 cell measured in the culture medium: (a) AFM topography, (b) deflection (“error”) images, and (c) 3D representation of a single alive melanoma WM266-4 cell measured in the culture medium.

**Fig. 2.2:**
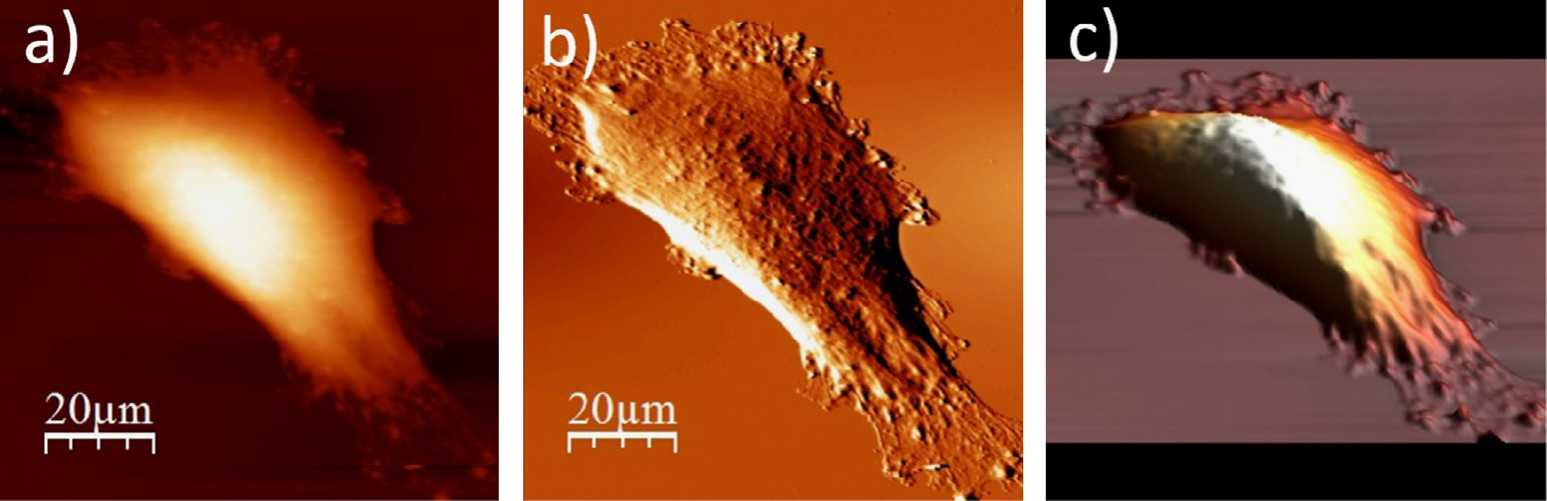
WM266-4 cell surface changes after fixation with paraformaldehyde: (a) AFM topography, (b) deflection (“error”) images, and (c) 3D representation of WM115 cell fixed with 3.7% of paraformaldehyde dissolved in the phosphate buffered saline (PBS) for 20 min, measured in the PBS buffer.

**Fig. 2.3:**
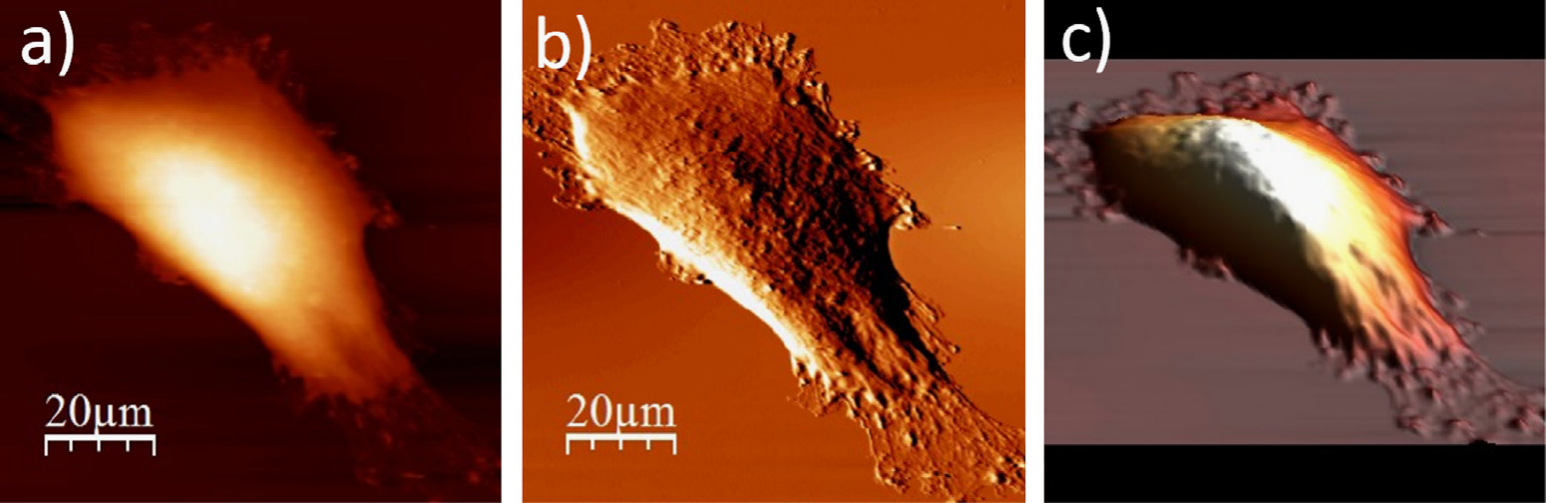
Surface of a single WM266-4 melanoma cell after rinsing in 50% PBS buffer: (a) AFM topography, (b) deflection (“error”) images, and (c) 3D representation of WM266-4 cell rinsed with 50% PBS buffer, measured in the 50% PBS buffer (1:1, PBS:water).

**Fig. 2.4:**
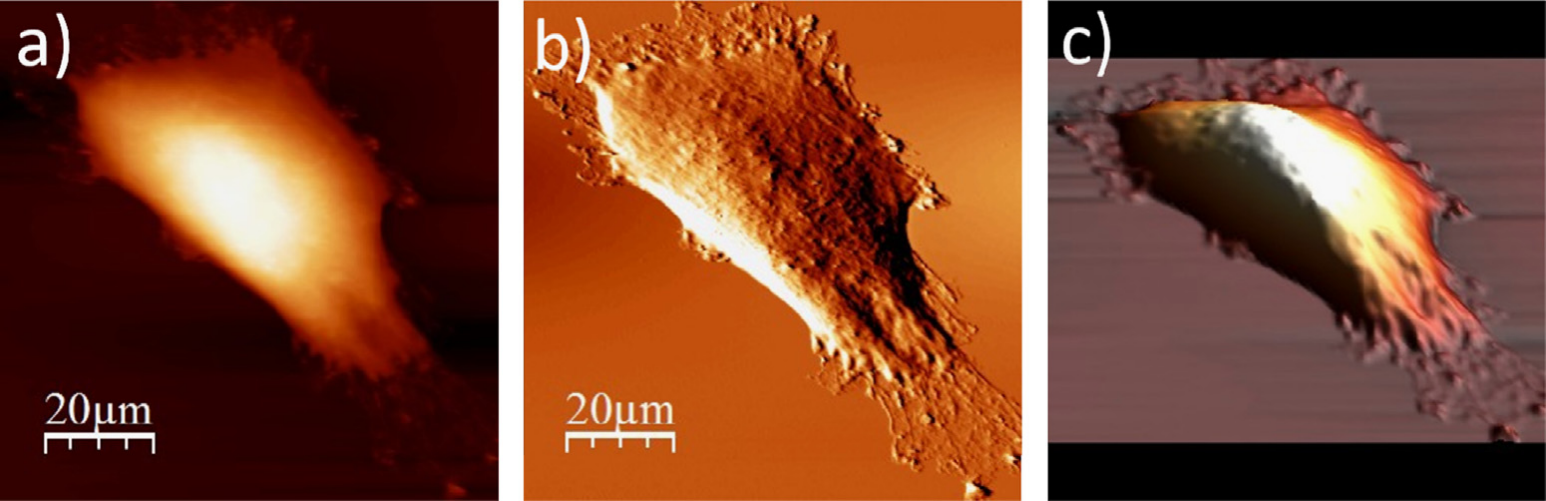
Surface of a single WM266-4 melanoma cell after rinsing in 25% PBS buffer: (a) AFM topography, (b) deflection (“error”) images, and (c) 3D representation of WM266-4 cell rinsed with 25% PBS buffer, measured in the 25% PBS buffer (1:4, PBS:water).

**Fig. 2.5:**
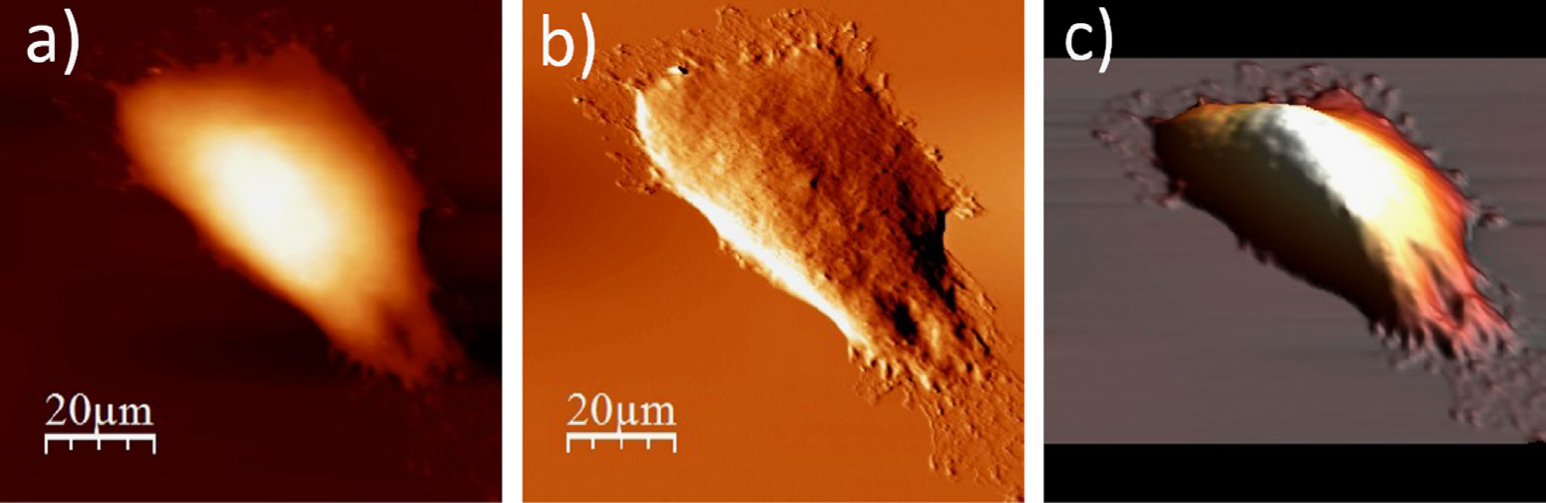
Surface of a single WM266-4 melanoma cell after rinsing with deionized water: (a) AFM topography, (b) deflection (“error”) images, and (c) 3D representation of WM266-4 cell rinsed with deionized water (Cobrabid purification system, 0.08 µS).

**Fig. 2.6:**
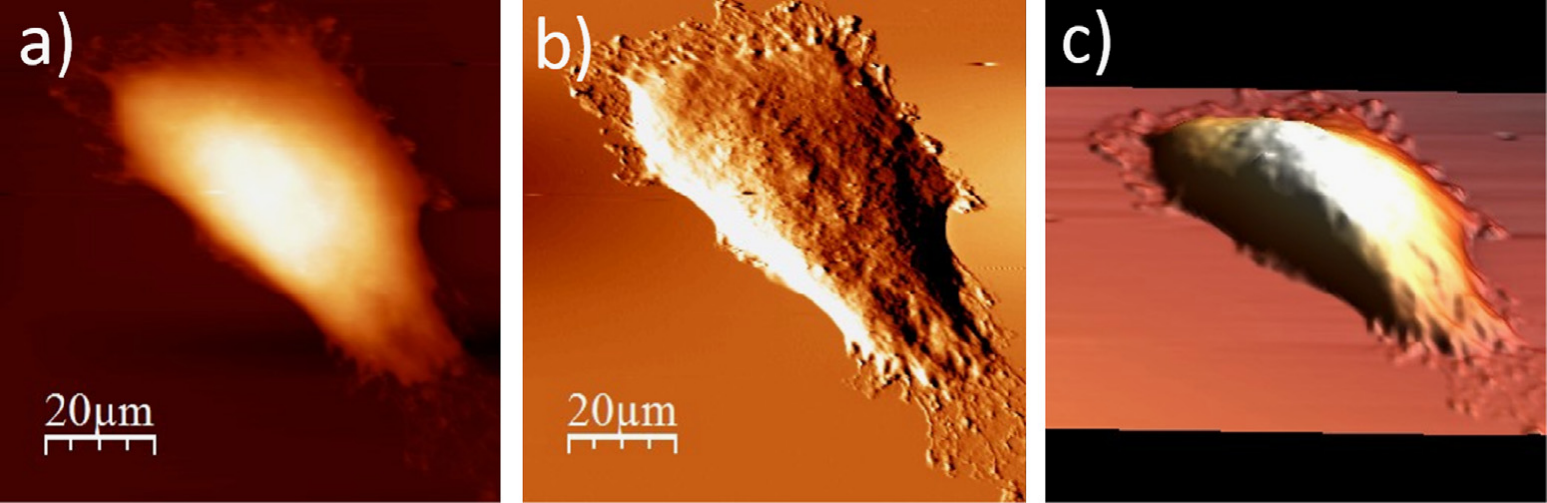
Surface of a single WM266-4 melanoma cell after rinsing with 40% alcohol: (a) AFM topography, (b) deflection (“error”) images, and (c) 3D representation of WM266-4 cell rinsed with 40% aqueous solution of ethyl alcohol.

**Fig. 2.7:**
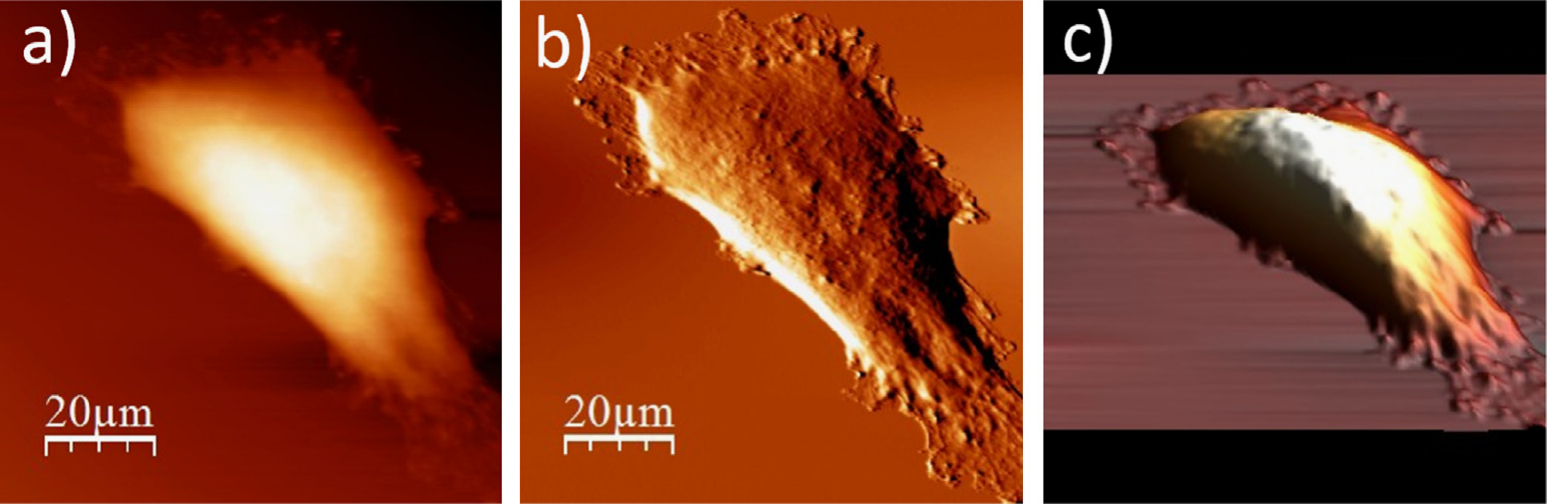
Surface of a single WM266-4 melanoma cell after rinsing with 50% alcohol: (a) AFM topography, (b) deflection (“error”) images, and (c) 3D representation of WM266-4 cell rinsed with 50% aqueous solution of ethyl alcohol.

**Fig. 2.8:**
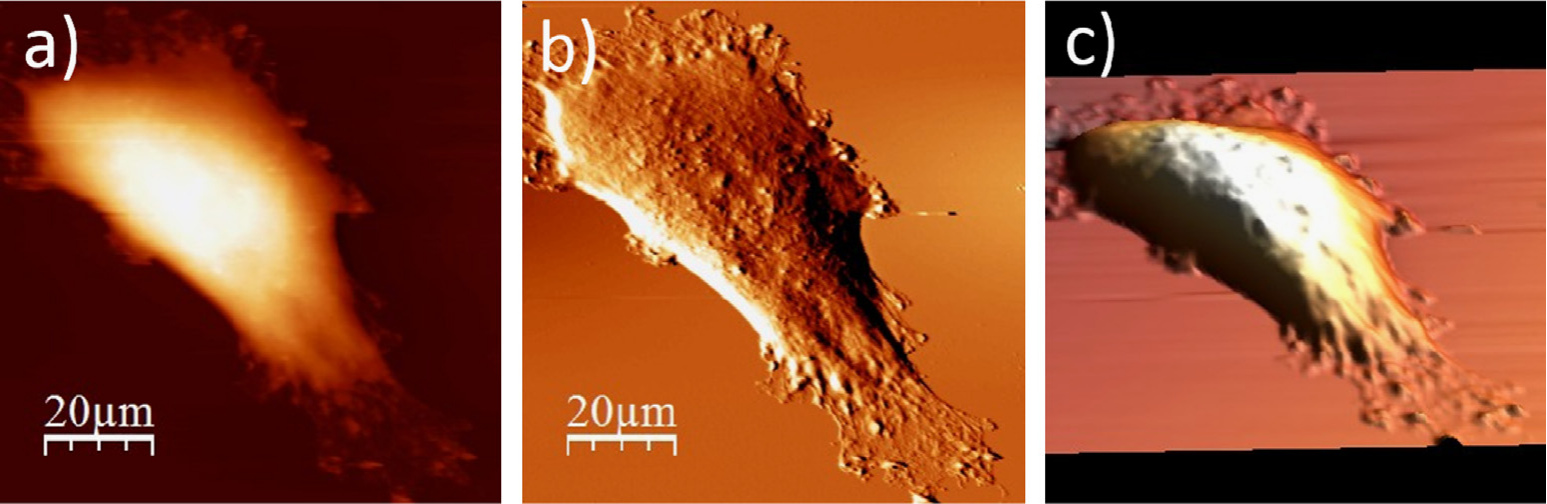
Surface of a single WM266-4 melanoma cell after rinsing with 60% alcohol: (a) AFM topography, (b) deflection (“error”) images, and (c) 3D representation of WM266-4 cell rinsed with 60% aqueous solution of ethyl alcohol.

**Fig. 2.9:**
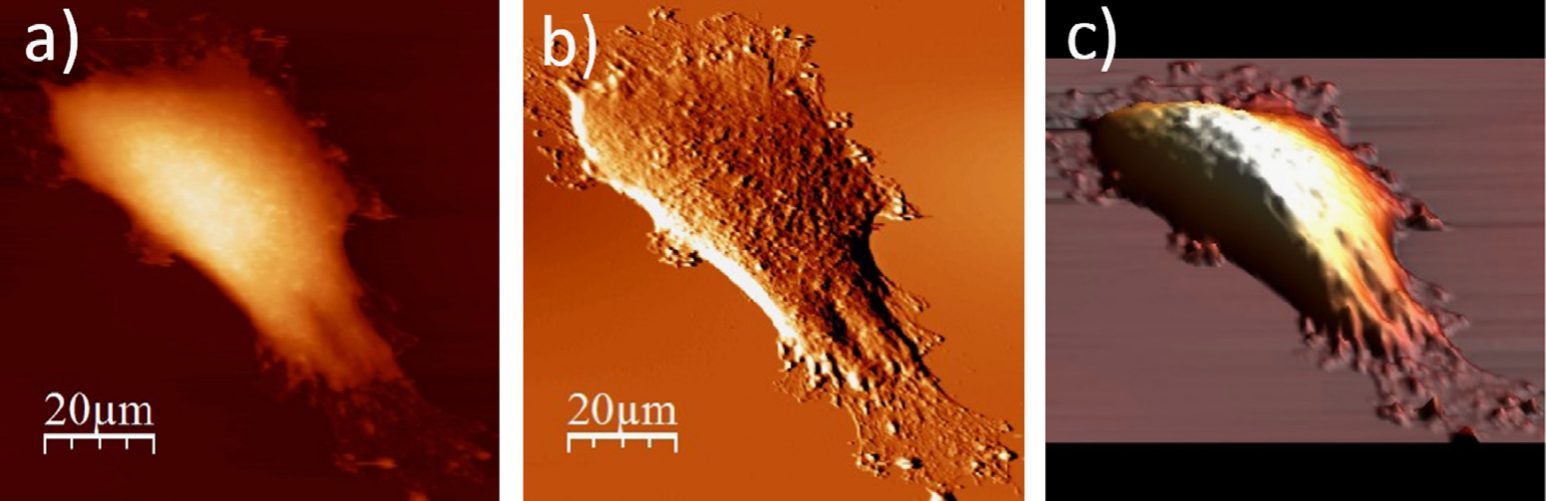
Surface of a single WM266-4 melanoma cell after rinsing with 70% alcohol: (a) AFM topography, (b) deflection (“error”) images, and (c) 3D representation of WM266-4 cell rinsed with 70% aqueous solution of ethyl alcohol.

**Fig. 2.10:**
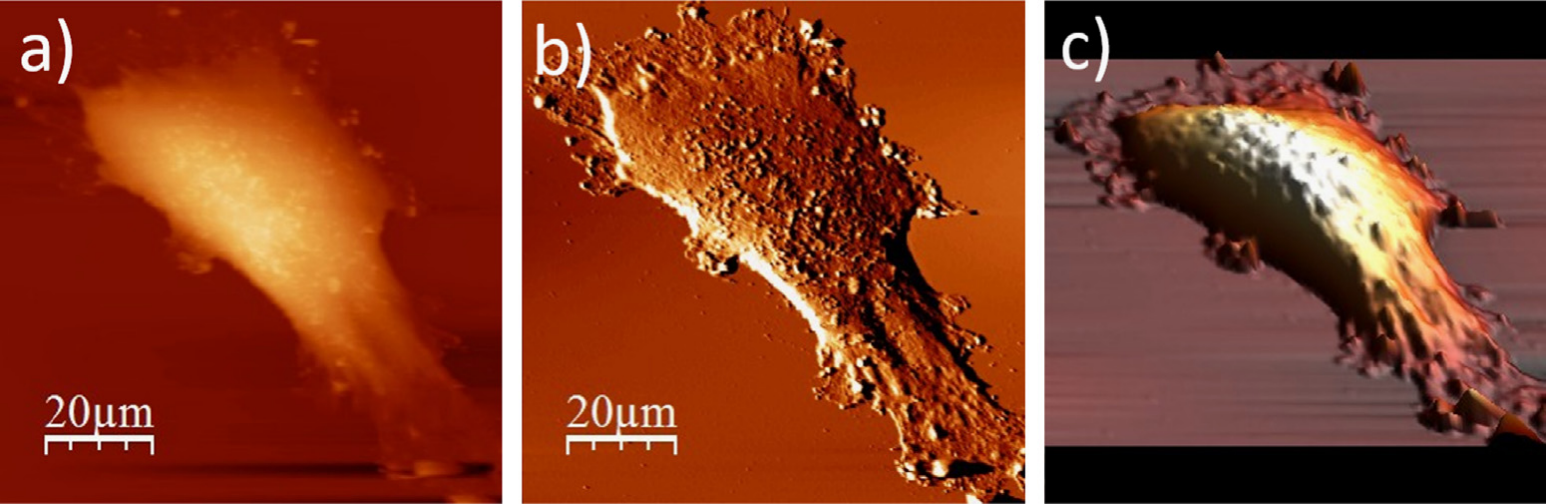
Surface of a single WM266-4 melanoma cell after rinsing with 80% alcohol: (a) AFM topography, (b) deflection (“error”) images, and (c) 3D representation of WM266-4 cell rinsed with 80% aqueous solution of ethyl alcohol.

**Fig. 2.11:**
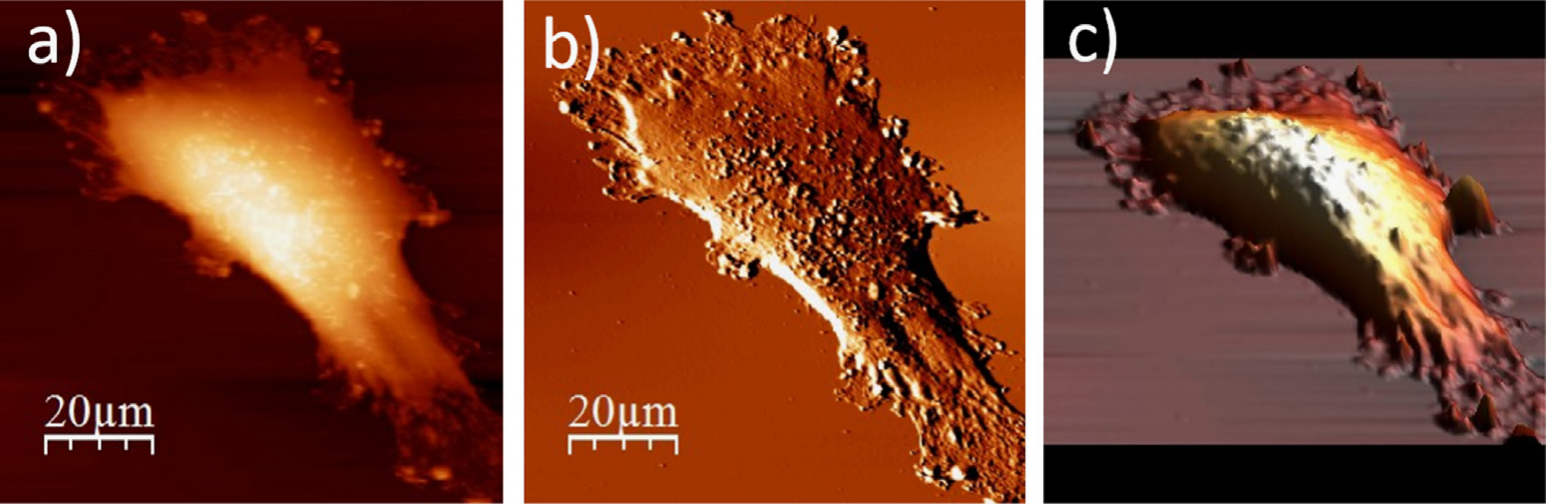
Surface of a single WM266-4 melanoma cell after rinsing with 90% alcohol: (a) AFM topography, (b) deflection (“error”) images, and (c) 3D representation of WM266-4 cell rinsed with 90% aqueous solution of ethyl alcohol.

**Fig. 2.12:**
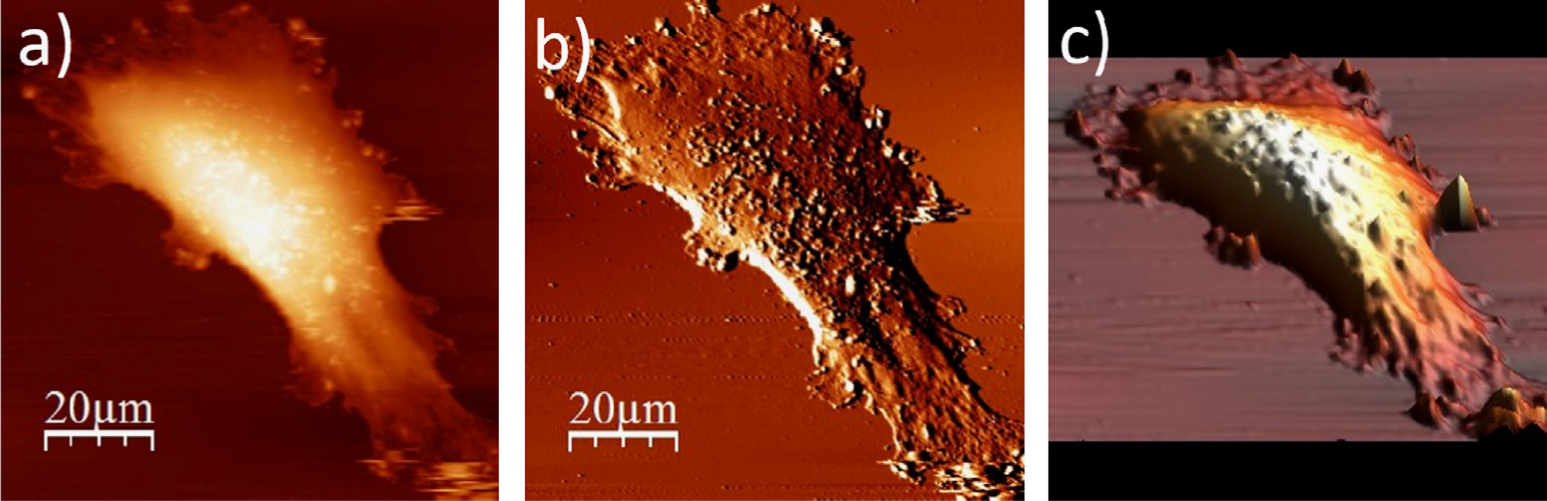
Surface of a single WM266-4 melanoma cell after rinsing with anhydrous alcohol: (a) AFM topography, (b) deflection (“error”) images, and (c) 3D representation of WM266-4 cell rinsed with anhydrous alcohol (99. 8%).

**Fig. 2.13:**
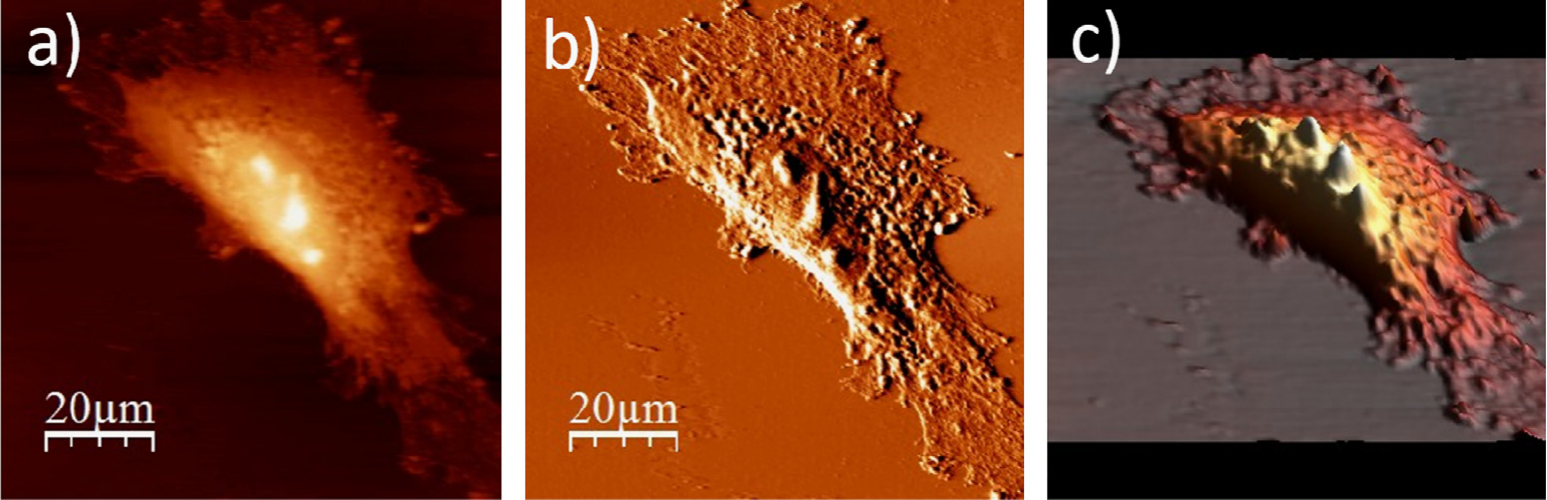
Surface of a dried single WM266-4 melanoma cell: (a) AFM topography, (b) deflection (“error”) images, and (c) 3D representation of a dried WM266-4 cell.
