# Supplementary material for: Data on step-by-step atomic force microscopy monitoring of changes occurring in single melanoma cells undergoing ToF SIMS specialized sample preparation protocol
Source: Data Brief. 2016 Aug 3;8:1322–32. doi: 10.1016/j.dib.2016.07.052 (PMC4990642; doi:10.1016/j.dib.2016.07.052)
Supplement: Supplementary file 1 — Supplementary material. [file mmc1.doc]

The authors declare no conflicts of interest.

Malgorzata Lekka
